# Automated Platform for the Analysis of Multi-Plate Growth and Reporter Data

**DOI:** 10.3390/microorganisms13081889

**Published:** 2025-08-13

**Authors:** Avichay Nahami, Dor Kain, Yonatan Cohen, Yuval Kolodkin-Gal, Yohanan Assouline, Avihu H. Yona, Ilana Kolodkin-Gal, Yuval Dorfan

**Affiliations:** 1 Faculty of Electrical and Electronics Engineering, HIT-Holon Institute of Technology, Holon 5810201, Israelyonhcohen@gmail.com (Y.C.);; 2 Scojen Institute for Synthetic Biology, Dina Recanati School of Medicine, Reichman University, Herzliya 4610101, Israel; 3 Institute of Biochemistry, Food Science and Nutrition, Robert H. Smith Faculty of Agriculture, Food and Environment, The Hebrew University of Jerusalem, Rehovot 7610001, Israel; avihu.yona@mail.huji.ac.il

**Keywords:** automation, data visualization, bacteriology, reporters, growth

## Abstract

Researchers traditionally calculate growth rates using the natural logarithm of optical density (OD), with existing script packages facilitating this process. Automatic plate readers, capable of simultaneously measuring OD across 384 cultures, significantly enhance data collection efficiency. Furthermore, these readers also measure luminescence and fluorescence, providing valuable insights into gene expression. However, current analysis software often struggle with data generated by robotic systems measuring multiple plates, limiting the integration of growth and reporter analyses. This method paper addresses three key challenges: (a) the incompatibility of robotic multi-plate systems with existing analysis software, (b) the integration of growth and reporter analyses, and (c) the development of user-friendly interfaces for non-programmers. To address these challenges, we offer optimized script packages and a relevant case study on matrix expression in response to antibiotics. Our platform facilitates the efficient and integrated analysis of multi-plate growth and reporter data.

## 1. Introduction

The analysis of microbial continuous monitoring assays frequently relies on growth rates, which have become increasingly valuable in microbiology for quantifying various phenotypic properties of microorganisms [1]. Measuring and analyzing growth has applications across multiple domains, including population dynamics and mathematical modeling [2,3]. In microbial sciences, growth rates serve as a crucial measurement of fitness and are used to assess the responses of microorganisms to different environmental factors, such as antibiotics [4]. To calculate growth rates, researchers often use the natural logarithm of the optical density (OD) of a cell culture over time [5]. Automatic plate readers can measure the OD of up to 384 cultures simultaneously, and the use of these instruments has greatly enhanced the efficiency of growth rate data collection. Furthermore, the software package GrowthRates 3.0 has revolutionized high-throughput analysis of growth rate data [5]. Growth Rate has been applied in various scientific fields, including the development of antibiotic cycling strategies to address antibiotic resistance [6], the creation of age phenotyping programs in yeast [7], monitoring bioactive products from antibiotic producers [8], comparing antibiotic resistance genes [9], and evaluating the response to plant exudates across generations [10].

In addition to measuring microbial growth, plate readers are commonly used to analyze various parameters, particularly luminescence, which can be measured simultaneously with OD [11]. Luminescence can provide insights into transcription from a transcriptional reporter or translation from a translational reporter, thus offering valuable layers of information alongside microbial growth [12].

The measurement of reporters in multiple assays is particularly important, given that the expression of genes with conditional patterns—such as stress-related genes and lifestyle-associated genes (e.g., biofilm formation and sporulation)—is not always correlated with microbial growth. In this methodology paper, we thoroughly address three features that have remained underdeveloped in previous studies: (a) many robotic systems designed to measure multiple plates simultaneously do not fit the existing software packages, (b) there is an increasing need to combine growth analysis with reporter analysis, a need that is often unaddressed, and (c) a user-friendly interface for non-programmers is required to facilitate the analysis.

The GROOT (Growth and Reporter Output Optimization Tool) package and examples of its optimal use are described throughout the paper. It can be used as an application or as an independent multi-layer code (Supporting Files S1 and S2). To illustrate potential applications, we analyzed the gene expression of a biofilm activator called SinI in the probiotic and biocontrol bacterium *Bacillus subtilis* [13]. We correlated this expression with growth during the antibiotic response, revealing non-linear relationships between growth and expression levels. In *B. subtilis*, Spo0A promotes biofilm formation by enhancing the production of SinI, a small protein that acts as an antagonist to the master repressor of biofilm formation, SinR [14]. SinR directly represses two operons involved in the matrix components, namely *epsA-O* and *tapA-sipW-tasA* [15,16,17]. Therefore, the transcription of SinI is the strongest predictor of biofilm gene activation in *B. subtilis*.

In this paper, we use the growth and reporter data of *B. subtilis* to showcase the output produced by GROOT software and its applications.

## 2. Materials and Methods

**Figure 1:**
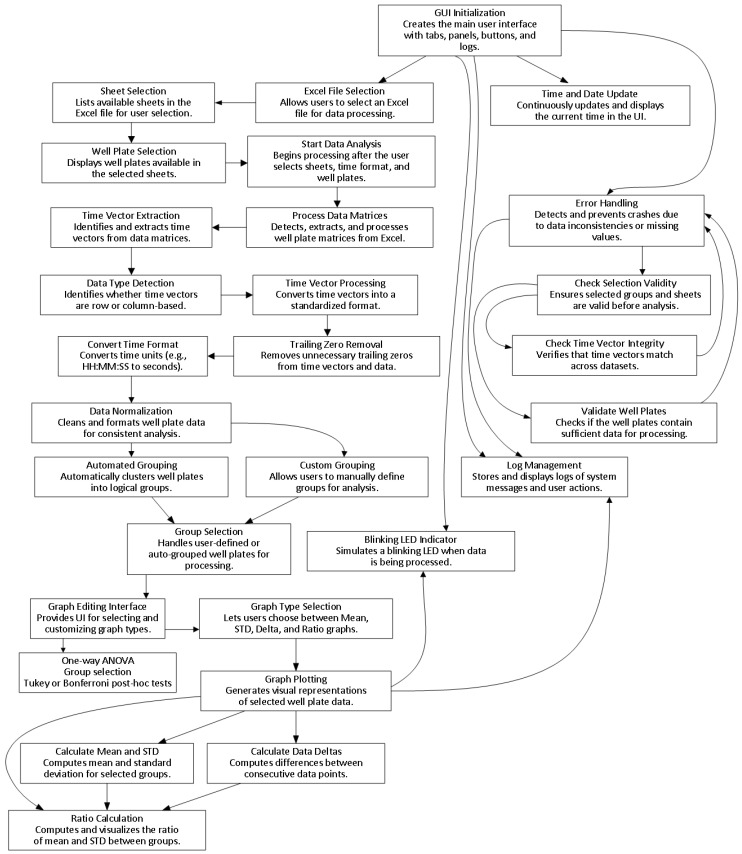
The GROOT (Growth and Reporter Output Optimization Tool) package and software design. Visualization of the GROOT architecture (see relevant text for full description and Supporting File S1 for the MATLAB script).

The *B. subtilis* strain used in this study is NCIB3610, which carries the *amyE::p_sinI_luciferase*: cam gene, IKbs0450 [18]. For all experiments, starter cultures were grown in LB Broth (BD Difco™ Dehydrated Culture Media: LB Broth, Thermo Fisher Scientific, Waltham, MA, USA). Cultures were diluted at a ratio of 1:50 in defined LB medium, with the desired concentration of kanamycin added to each well of a 96-well polystyrene Visiplate (Wallac), which was white with a clear bottom. The cultures were then grown for 30 h at 30 °C in a Tecan Infinite 200 Pro plate reader (Tecan, Männedorf, Switzerland). We verified the experiment and the tool in two other plate readers, BioTek LogPhase 600 Microbiology reader and BioTek Synergy H1 Multimode Reader (Agilent Technologies, Santa Clara, CA, USA), and the optical density at 600 nm (OD600) and luminescence (with a sensitivity setting of 200) were measured every 10 min. The data in the figures represent averages from four replicate wells of a single representative experiment, where luminescence values were normalized to the highest recorded luminescence. All data were analyzed using the GROOT package, as shown in Figure 1. The GROOT software accommodates all potential measurements conducted with a plate-reader, regardless of the type of bacterium, treatment, or readout (OD, luminescence, color development, fluorescence).

## 3. The Architecture of GROOT Software

### 3.1. General

The architecture of GROOT is described in Figure 1 and in Supporting Figure S1. The MATLAB file is provided in Supporting File S1, and the instructions file is provided as a Supporting Readme File (Supporting File S2).

Plate Reader Analyzer (MATLAB v 13.0) is architected as a layered event-driven application that converts raw multi-well plate-reader exports into statistically annotated graphics through a single-window GUI. Its design follows a strict separation of concerns: (i) a data-ingest layer that discovers and cleans matrices, (ii) a model layer that hosts normalized time–signal objects and logical groupings, and (iii) a presentation layer—two coordinated tab panels—that renders interactive plots and handles user commands. All inter-layer communication is mediated via setappdata/getappdata, guaranteeing persistence without global state leakage.

### 3.2. Data-Ingest Layer

The data layer includes four components:

1. Automated block discovery: Each selected Excel sheet is parsed once. A vectorized search for characteristic matrix header starts with coordinates; this permits arbitrarily located blocks and mixed orientations in a single workbook.
2. Orientation and integrity checks: The helper DetermineVectorType classifies matrices as vertical- or horizontal-time, after which empty margins, NaN seams, trailing zero segments, and Excel’s > 24 h rollover are excised. Misaligned time vectors (>180 s) are rejected early, raising non-blocking warnings via the logging service.
3. Canonicalization: Time stamps are coerced to seconds, re-exported in user-chosen units, and welded to numeric payloads whose columns represent wells. Text labels are id-prefixed (MX01–MXnn) to remain unique across sheets.
4. The resulting objects: Time, data, and label form the model layer.

### 3.3. Model Layer

The model layer is the first processing step. It contains two components:

1. Hierarchical grouping: A dual-tree structure stores native groups (sheet→well) and selected groups (Z01–Z99), or automatic groups by the plate letters (A1-A12→MXnA). The mover widget clones checked well into custom parents without mutating source nodes; this is implemented as lightweight handle copies, that is, memory scales with selections, not data size.
2. Derived statistics: Vectorized routines expose on-demand views: mean ± SD envelopes, first differences, and propagated ratio errors. All statistics preserve NaN positions, ensuring downstream plots reflect missingness transparently.

### 3.4. Calculations Layer

Once each group stores a numeric matrix *D*(*n_wells_* × *n_t_*) and matching time vector *t*, a vectorized kernel derives all downstream statistics in a single pass: the mean trace *μ*(*t*) = mean(*D*,1), the standard deviation envelope *σ*(*t*) = std(*D*,0,1), and for kinetic emphasis, the first difference in every well $\Delta D_{i}\left(t\right)=diff\left(D_{i}\right)$, followed by its group mean $\mu_{\Delta }(t)$ and SD $\sigma_{\Delta }(t)$. For pairwise comparison, the engine computes the point-wise ratio of two groups’ means

(1) $$\rho \left(t\right)=\mu_{1}(t)/\mu_{2}(t)$$

with propagated uncertainty.

(2) $$\sigma_{\rho }\left(t\right)=\rho (t)\sqrt{{\left[\sigma_{1}(t)/\mu_{1}(t)\right]}^{2}+{\left[\sigma_{2}(t)/\mu_{2}(t)\right]}^{2}}$$

All routines omit NaNs yet keep their positions, ensuring gaps remain explicit in plotted traces. Binary operations first verify temporal congruence; if max|*t*1 − *t*2| > 180 s, the calculation is aborted, and a non-blocking warning is logged. The resulting statistic objects are immutable and cached in the model layer, so the presentation layer can render any view without re-computation.

### 3.5. Presentation Layer

The GUI interface consists of three tabs in the following order:

1. Setup tab: Orchestrates file selection, sheet list, time-unit dropdowns, and ingest progress. A blinking uilamp is toggled by a timer that is instantiated and deterministically destroyed inside analyzeData, preventing orphan threads.
2. Graph-editing tab (selected manually): Radio-button groups choose the visual mode. Dropdowns directly connect to live group lists via observers, Edit Graph Labels button enables customization of the axis and main title labels, and legend box can be modified manually. Plot routines share a single UI axes object, cleared only when the user requests a reset, allowing multilayer storytelling. Custom data tips and HSV/greyscale palettes enhance traceability.
3. Audit trail: All status messages funnel through custom_fprintf—which has timestamps and color-coded errors—and stream simultaneously to a pop-up log window and in-panel label.

By integrating discovery, canonicalization, statistical modeling, and rich visualization behind a minimal GUI, Plate Reader Analyzer delivers a reproducible, crash-resistant pipeline that transforms heterogeneous plate-reader spreadsheets into publication-ready kinetic analyses with zero manual scripting.

### 3.6. Statistics

For each group i (i =1,…,k) with $n_{i}$ wells and response values $Y_{ij}$, total variability is partitioned into between-groups and within-groups sums of squares:

(3) $$\begin{array}{c} SS_{\mathrm{Groups}}=\sum_{i=1}^{k}n_{i}{\left({\bar{Y}}_{i.}-{\bar{Y}}_{..}\right)}^{2},\;df_{\mathrm{Groups}}=k-1\\ SS_{\mathrm{Error}}=\sum_{i=1}^{k}\sum_{j=1}^{n_{i}}{\left(Y_{ij}-{\bar{Y}}_{i.}\right)}^{2},\;df_{\mathrm{Error}}=N-k, \end{array}$$

where $N\;=\sum_{i\;=1}^{k}n_{i},{\bar{Y}}_{i}$. is the mean of group i and ${\bar{Y}}_{..}$ is the grand mean. Mean squares follow by division and the F-ratio used by the GUI is

(4) $$MS=\frac{SS}{df},\;F=\frac{MS_{\mathrm{Groups}}}{MS_{\mathrm{Error}}}∼F_{k-1,N-k}$$

The ANOVA tab reports SS,df,MS,F, and the associated p-value; when F is significant at α =0.05 it enables Tukey–Kramer and Bonferroni multiple-comparison tests via the Show post hoc plot button.

GROOT is available as a free code as following: https://github.com/dorkain22/GROOT/tree/main (accessed on 7 August 2025).

## 4. Results

### 4.1. GROOT User Guide

We developed a user-friendly package with the following functions: (a) creation of a main user interface, (b) clear selection of data from multiple wells on a single plate or across multiple plates, and (c) performance of analysis functions related to growth, reporter analysis, or both. The script is provided as Supporting File S1 (Plate_Reader_Analysis_ver14_1.m).

Using this platform, we analyzed the growth of a strain containing a transcriptional reporter for the activity of the *sinI* promoter at various concentrations of the antibiotic kanamycin [19]. This aminoglycoside inhibits protein synthesis by binding to the 30S subunit of the bacterial ribosome and is a broad-spectrum antibiotic effective against both Gram-positive and Gram-negative bacteria [19]. To monitor the activity of the *sinI* promoter in real time, we utilized a single copy P_sinI_-luciferase fusion and measured light production using a luminometer in a plate reader.

### 4.2. Growth and Reporter Data Analysis

**Figure 2:**
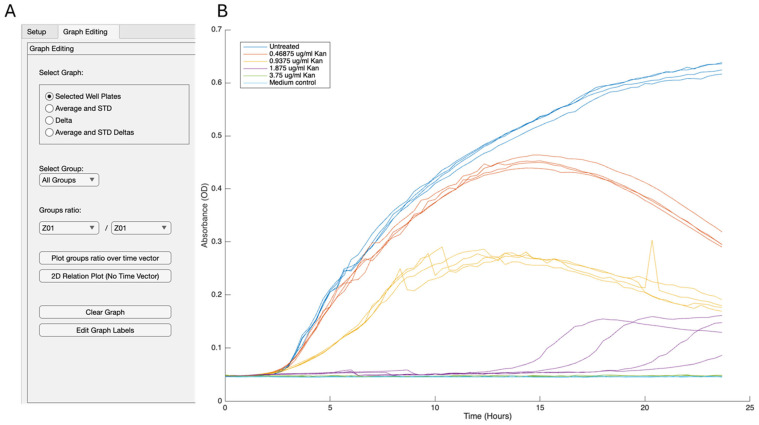
Growth analysis of individual repeats. The growth of *B. subtilis* in LB was measured every 30 min with optical absorbance (OD600). We measured either untreated cultures or cultures that were applied with different concentrations of kanamycin and analyzed them with GROOT software. (**A**) The parameters used to generate the figure. (**B**) Each well is represented separately in the graph.

**Figure 3:**
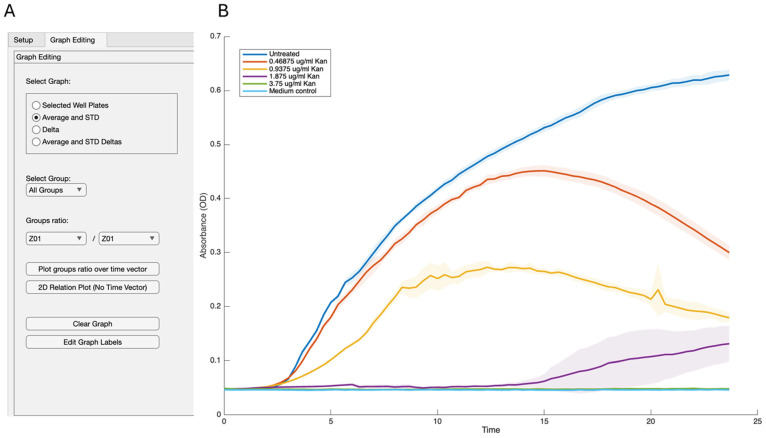
Growth analysis of the experiment. The growth of *B. subtilis* in LB was measured every 30 min with optical absorbance (OD600). We measured either untreated cultures or cultures that were applied with different concentrations of kanamycin and analyzed them with GROOT software. (**A**) Parameters used to generate the figure. (**B**) GROOT-generated figure. Results are the average and standard deviation of four technical repeats. The standard deviation is represented in halo.

We created several layouts to illustrate different aspects of growth. One layout highlights each group’s average and standard deviation, providing a smooth transition from the Excel file. This representation clearly shows the impact of antibiotics on bacterial growth, including the dose response and its effect on the growth curve itself (Supporting Files S3 and S4). Growth is shown as an OD_600_ versus time. To better evaluate outliers and variations within the group, we can easily create a representation of each well overtime (Figure 2) or show the average of all individual repeats with their standard deviations (Figure 3). Notably, for growth rates, the rate, carrying capacity, and length of the lag phase can be easily assessed. Therefore, we did not focus on this application in detail. These results demonstrate minimal noise while assessing the growth of *B. subtilis* under all tested conditions. There is some increase in the heterogeneity level between the technical repeats at concentrations that are significantly inhibitory to growth, under our conditions, 1.8 µg/mL.

**Figure 4:**
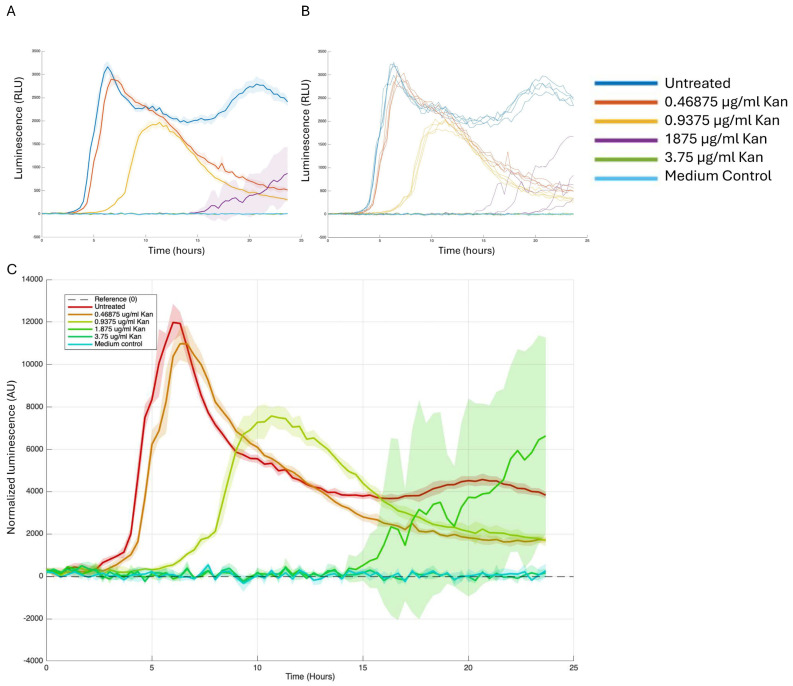
The analysis of reporter data by Groot. Strain 3610 carrying the *sinI* luminescence reporter was grown in either LB medium (NT) or LB medium supplemented with the relevant indicated kanamycin concentration. (**A**) A 96-well plate with white opaque walls and clear tissue culture-treated flat bottoms (Corning, New York, NY, USA) was used for the measurements. Measurements were performed every 30 min at 30 °C, using a microplate reader (Synergy 2; BioTek, Winooski, VT, USA). (**A**) Results are the average and standard deviation of four technical repeats. The standard deviation is represented in halo. (**B**) Each well is represented separately in the graph. (**C**) Luciferase activity was calculated as RLU/OD. Growth was monitored to avoid artifacts related to the normalization of luminescence intensity to the population size. For presentation purposes of the multipaneled figures, the legend was manually enlarged.

We then assessed our signal, specifically the luminescence that indicates biofilm gene expression. We created a normalization step to address variations in signal intensity caused by differences in bacterial numbers. This involved developing a script that adjusts luminescence measurements based on growth, like approaches used by others and us in previous studies [14,16]. We took several independent approaches, showing bulk luminescence as average and standard deviation of the group or as separate wells (Figure 4A,B), and showing normalized luminescence on the *Y*-axis, which is calculated as luminescence per OD (Figure 4C).

The results shown in Figure 4 demonstrate a significant reduction in transcription at concentrations starting from 0.9 µg/mL, which did not impact cell growth. Non-toxic concentrations of kanamycin not only reduced the peak normalized intensity of the reporter but also influenced the timing of its initial activation prior to affecting bacterial growth. This straightforward application of the script enables the visualization of standard deviation, represented as a halo in Figure 4B,C for bulk and normalized intensity. It is important to note that this approach can be applied to various time-resolved measurements, including colorimetric, transcriptional, or translational reporter assessments, such as fluorescence or pigmentation.

**Figure 5:**
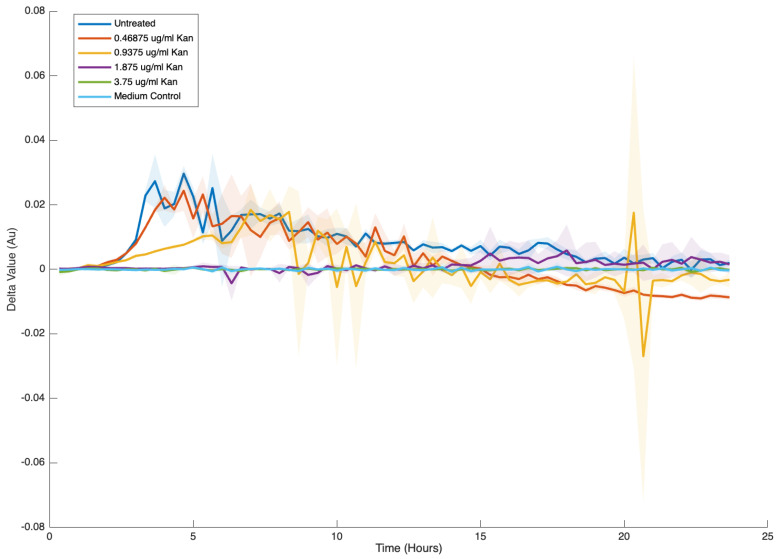
Analyzing the differences between measured time points. The delta between single growth measurements over time (Figure 2) is represented following analysis with the GROOT software. Results are the average and standard deviation of four technical repeats. The standard deviation is represented in halo.

We also explored a non-conventional approach to monitoring changes in the reaction rate. While the assessment of the rate, as reflected by the slant, is commonly used, we found it beneficial to plot the difference between subsequent points. Although short time intervals between measurements may not allow for a significant evaluation of change, consistent monitoring of value fluctuations helps identify transitions between phases of induction and decay of a relevant property (such as growth or normalized transcription) over time. This method provides clear indications of the emergence of a death stage, which is reflected in a continuous and consistent drop in the curve’s values, specifically when the curve descends below zero on the relevant axis (Figure 5).

### 4.3. Statistical Analysis Features of GROOT

The one-way ANOVA tab lets users test whether any custom or native groups differ significantly at specific, user-selected time points (s). On the left, a multi-select list box shows all defined groups, and beneath it, a factor name field (e.g., “Optical Density”) and a time point (s) for ANOVA selector list, every time stamp common to all groups, defaulting to the final one. The user may highlight one- or several-time values, then choose Tukey or Bonferroni from the post hoc dropdown. Pressing Run ANOVA extracts each well’s measurement at the chosen time (s), averages them if more than one is selected, and performs a one-way ANOVA that tolerates unequal group sizes. The tab fills a results table with SS, df, MS, F, and *p*-values; a color-coded box-and-whisker plot of the same data appears below, its title echoing the selected time (s) and *p*-value for instant interpretation (See Figure S2). If a post hoc test is requested, the Show post hoc plot button activates, launching MATLAB’s multiple-comparison window so pairwise differences can be inspected visually.

#### Data Export and Housekeeping

The Export results table button writes the ANOVA summary to.csv, while Clear ANOVA purges the table, plot, and cached statistics, returning the tab to a blank state. Status messages—successful export, missing time points, etc.—continue to funnel through the interface’s audit-trail label, ensuring a consistent user experience.

### 4.4. Growth-Expression Dynamics

**Figure 6:**
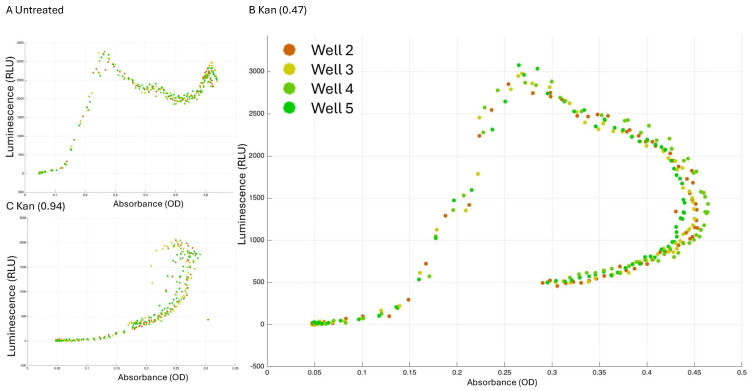
Reporter data analysis. Strain 3610 carrying the indicated luminescence reporter was grown in either LB medium (NT) or LB medium supplemented with the relevant indicated kanamycin concentration. (**A**) A 96-well plate with white opaque walls and clear tissue culture-treated flat bottoms (Corning) was used for the measurements. Measurements were performed every 30 min at 30 °C, using a microplate reader (Synergy 2; BioTek, Winooski, VT, USA). The non-normalized luminescence of P_sinI_ reporter in LB applied with different concentrations of kanamycin is plotted versus OD600 (growth) with GROOT software. Measurement of each well is represented separately. (**A**) Untreated, (**B**) kanamycin 0.47 µg/mL, (**C**) kanamycin 0.94 µg/mL. For presentation purposes of the multipaneled figures, the legend was manually enlarged.

One method to easily correlate growth and transcription activation is to plot the expression as a function of measured OD (e.g., two separate measurements in separate Excel spreadsheets to be plotted against each other, correlating data from the same well). Figure 6 represents the transcription from P*_sinI_* as a function of growth stages. Results indicate the intriguing pattern of expression of this de-repressor, consistent with previous studies [16] as it increases at mid-log and then picks up again during the stationary phase (Figure 6A).

It is particularly interesting to observe the unnormalized expression of this reporter in transcription while regulating kanamycin concentrations (0.47 and 0.94 µg/mL, Figure 6B,C). These results emphasize the significance of plotting transcription against OD600 (growth parameters) instead of solely depending on normalized data to evaluate transcription dynamics. Notably, these findings reveal that the bacterial biomass at a specific time point is inadequate for accurately predicting the transcription of the biofilm activator. Furthermore, the dynamics and regression of transcription vary significantly across different concentrations, indicating that *sinI* transcription reflects microbial history. In this study, the expression of *sinI* is explored solely as a case study, indicating that this is a correlation rather than causality, which warrants additional in-depth research.

**Figure 7:**
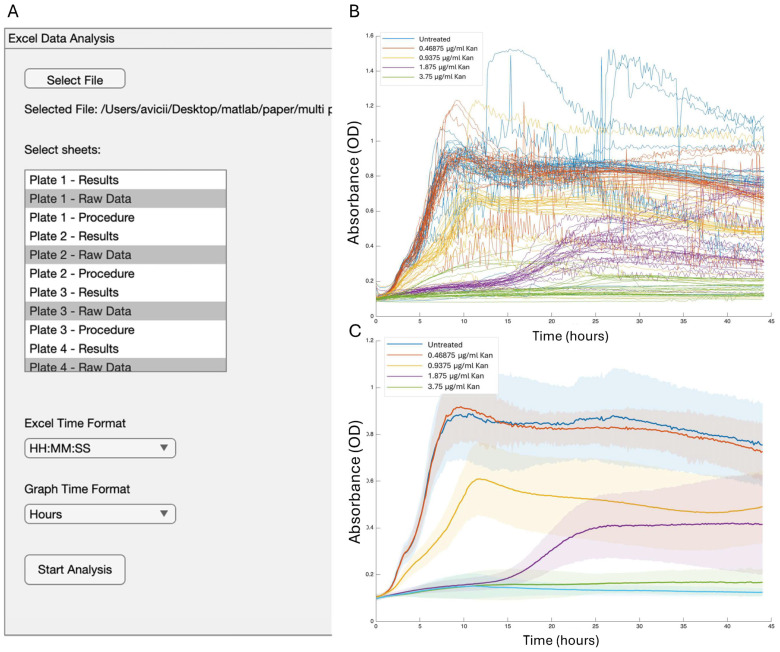
Analyzing robotic measurement of multiple plates simultaneously. The growth of *B. subtilis* 3610 in LB applied with different concentrations of kanamycin analyzing with GROOT software. (**A**) Parameters used to generate the figure. (**B**) The GROOT output for growth analysis with single well presentation. (**C**) GROOT-generated figure. Results are the average and standard deviation of four technical repeats. The STDEV is represented in halo. For presentation purposes of the multipaneled figures, the legend was manually enlarged.

Lastly, we utilized our software on a robotic multi-plate reader capable of reading four plates simultaneously. For this purpose, we generated an architecture capable of integrating the data from multiple plates measured simultaneously (Figure 7A,B, Supporting Files S5 and S6). Integrating the data to calculate the average and standard deviation enabled the creation of coherent figures (Figure 7C), allowing for a quick evaluation of the data and streamlining the analysis of multiple plates. The results were consistent with measurements taken from individual plates (Figure 2 and Figure 3).

## 5. Discussion

Bacterial culture growth is a foundational method in microbiology. While the emergence of molecular tools has introduced new research opportunities, it is essential to continue exploring growth studies as a key technique. The complexity of this growth process, involving various interconnected mechanisms, can be effectively captured using mathematical models, and several effective tools have been developed to extract data efficiently. However, there is an emerging importance of generating reporter data analysis to match microbial growth, and for clear and reasonable presentation platforms for multiple and multi-well assays. The unique features of GROOT versus similar applications are detailed in Supporting Table S1, indicating clear advantages for flexibility and a user-adaptable interface.

The genetic reporter assay is routinely used to investigate the interplay between DNA sequences and their regulatory activities. The traditional applications of this assay have faced challenges due to the necessity of individual cloning and analyzing each sequence of interest, with protein fluorescence or luminescent activity being used as indicators. However, recent advancements in high-throughput DNA synthesis and sequencing technologies present efficient systems and synthetic biology solutions. These innovations allow for the multiplexing of the construction and interrogation of extensive libraries of reporter constructs, significantly enhancing throughput and efficiency.

Our protocol allows quick and efficient tools to screen phenotypes and assess their relation to growth, allowing a comprehensive understanding of reporter growth interactions and their complexity. The example of a biofilm activator being fully independent of OD with antibiotic treatment and altering its growth-production kinetics as a function of cell history is described here in Figure 6 as a strong example of our approach. This analysis (Figure 7) can assist high-throughput screens in single-well and multi-well assays as well as automatic and user-friendly analysis platforms. In the future, this tool can also serve as the pre-processing step for sophisticated analysis, including AI-driven approaches.

## Data Availability

The original contributions presented in this study are included in the article/Supplementary Materials. Further inquiries can be directed to the corresponding authors.

## Supplementary Materials

The following supporting information can be downloaded at: https://www.mdpi.com/article/10.3390/microorganisms13081889/s1. Supporting Table S1: GROOT Characterization. Supporting File S1: PDF file with the GROOT script usage manual. Supporting File S2: Word file with the instructions for downloading the script from Github. Supporting File S3: Excel file of the plate reader data used to generate Figure 2, Figure 3, Figure 4, Figure 5 and Figure 6. Supporting File S4: Excel file of well classification of the experiment included in Supporting File S3. Supporting File S5: Excel file of the plate reader data collected for multi-plate and used to generate Figure 7. Supporting File S6: Excel file of well classification of the experiment included in Figure 7 with data provided in Supporting File S5. Supporting Figure S1: Four mandatory steps of utilizing the GROOT application. Supporting Figure S2: (A) GROOT user interface for statistical analysis. (B) ANOVA analysis of all groups at a given time point. The table was generated with ChatGPT o4 Plus software and was cured by AN and YKG based on user-experience and feedback.
