# Supplementary material for: Automated Platform for the Analysis of Multi-Plate Growth and Reporter Data
Source: Microorganisms. 2025 Aug 13;13(8):1889. doi: 10.3390/microorganisms13081889 (PMC12388276; doi:10.3390/microorganisms13081889)
Supplement: Supplementary file 1 [file microorganisms-13-01889-s001.zip › microorganisms-3699852-supplementary.pdf]

Supporting Files.

Supporting Table S1- GROOT Characterization

Supporting File S1 - PDF file with the GROOT script usage manual

Supporting File S2 - Word file with the instructions for downloading the script from github

Supporting File S3- Excel file of the plate reader data used to generate Figures 2-6

Supporting File S4 - Excel file of well classification of the experiment included in supporting file 03

Supporting File S5- Excel file of the plate reader data collected for multiplate and used to generate figure 7

Supporting File S6 - Excel file of well classification of the experiment included in supporting file 05

Supporting Figure S1

Supporting Figure S2

Software	Selective Wells	Horizontal/Vertical Data	Multi-reader Support	Normalize Lum (OD/Lum)	OD vs Lum Scatter	Statistical Analysis	Usage Cost
Groot	Yes	Yes	Yes	Yes	Yes	Advanced	Free
SoftMax Pro 7	No	Yes	Partial	Yes	No	Basic	Paid
SpectraMax L	No	Yes	No	No	No	Basic	Paid
BMG Voyager/ I-Control	No	Yes	Yes	Yes	Limited	Advanced	Free w/ Instrument
BMG LUMIstar Omega	No	Yes	No	No	No	Moderate	Free w/ Instrument
BMG PHERAstar FSX	No	Yes	No	Yes	Partial	Advanced	Free w/ Instrument
Tecan Magellan	No	Yes	Yes	Yes	No	Advanced	Paid
Agilent BioTek Gen5	No	Yes	Yes	Yes	No	Moderate	Free (Standard) Paid (Secure)
PlotXpress	No	Yes	Yes	Yes	No	Basic	Free

Supporting Table S1. GROOT characterization versus existing similar software and codes

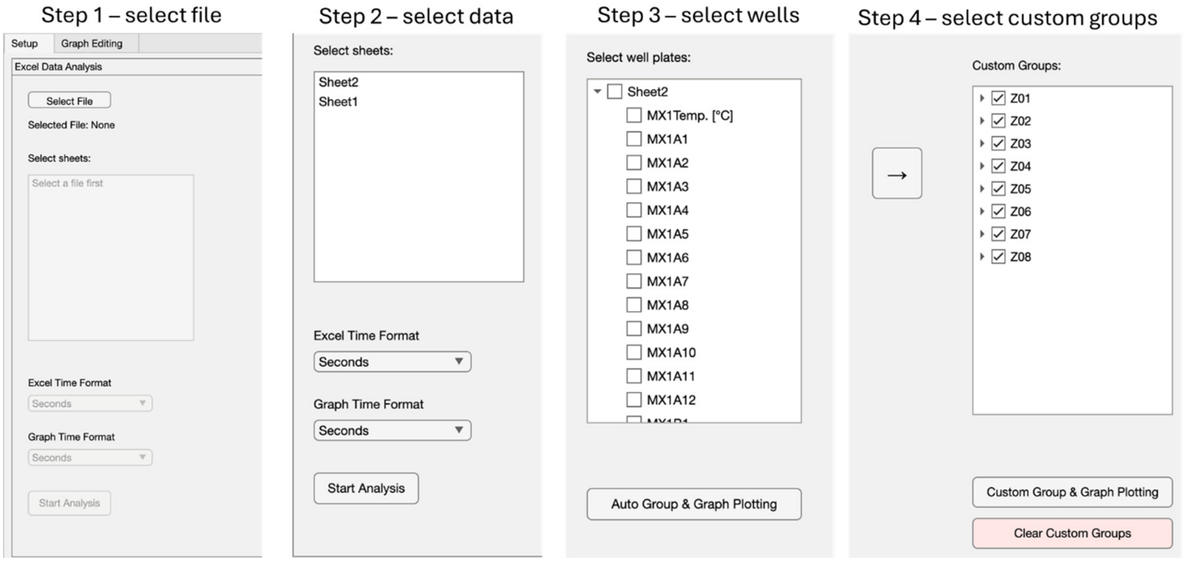

Figure S1: Four mandatory steps of utilizing the GROOT application

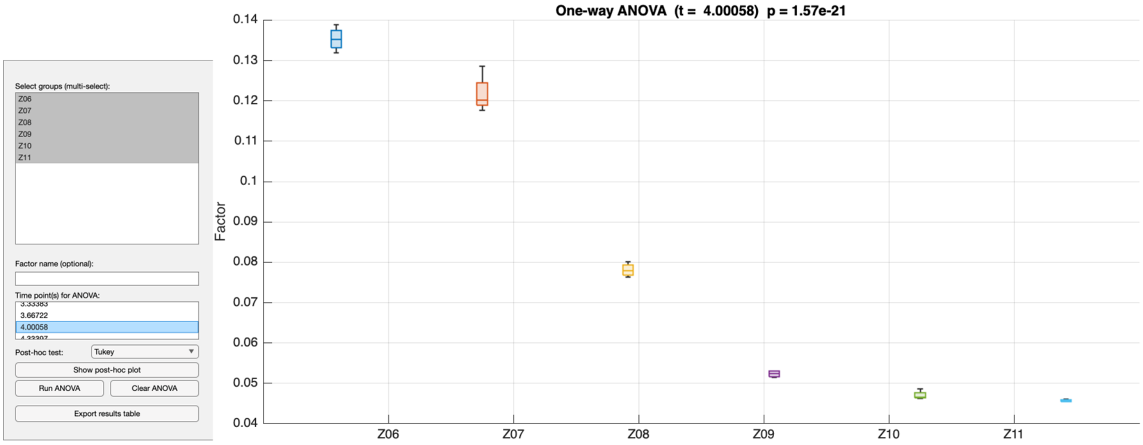

Figure S2: A. GROOT user interface for statistical analysis. B. Anova Analysis of All Groups at a Given Time Point. The table was generated with ChatGPT o4 Plus software and was cured by AN and YKG based on user-experience and feedback.
