# Supplementary material for: Automated Platform for the Analysis of Multi-Plate Growth and Reporter Data
Source: Microorganisms. 2025 Aug 13;13(8):1889. doi: 10.3390/microorganisms13081889 (PMC12388276; doi:10.3390/microorganisms13081889)
Supplement: Supplementary file 1 [file microorganisms-13-01889-s001.zip › supporting 01.pdf]

Link for github: https://github.com/dorkain22/GROOT/tree/main

MATLAB code guide:

MATLAB code guide:

1 Open the code in MATLAB.

2 Press "Run" to start the code.

3 Wait for the software code to upload.

4 Press "Select File" and choose the file you want to analyze.

*Take action in the left box "Select sheets.":

5 Choose the sheet that contains your raw data.

*The user can choose multiple sheets to analyze.

6 Select the right excel time format in the "Excel Time Format" options .

7 Select the format of the desired time frame for X-axis in your graphs in "Graph Time Format."

8 Press the button "Start Analysis"

*Take action in the middle box "Select well plates.":

*this part is for selecting groups to compare each other.

*MXn - n refers to the order of the matrix in the selected excel sheet.

" *Auto Group & Graph Plotting" can be use if your sample group is in the letters of the well plate (all A - group 1, all B - group 2, ... all H - group 8)

*If you want to analyze luminescence also choose their groups.

9 Selected the desired wells by press their box.

10 After selecting all the wells for the first group press the arrow sign between the middle and the right box.

11 Repeat step 9-10 as many as you need.

*This part takes action in the right box "Custom Groups":

12 Choose the desired groups for further analysis by marking them.

13 Press the "Custom Group & Graph Plotting."

*If you make a mistake you can clean the right box "Custom Groups:" by pressing "Clear Custom Groups."

*First, only choose the OD data, after finish make graphs of the OD data mark also the luminescence data and press "Custom Group & Graph Plotting" again.

*The next steps will perform actions in the "Graph Editing" in the top left tab in the software tab by click on him.

*In "Graph Editing" you have multiple analysis option.

14 Selected Well Plates" - will show you in the graph all the wells individually but there are colors to indicate their groups.

15 Average and STD" - will show you the average and std of the selected groups.

16 "Delta" - will show you all the delta values individually but there are colors to indicate their groups.

17 "Average and STD Delta" - will show you average and std of the delta values of the selected groups.

*In "Select Group:" option you can choose a specific group to focus on, if you want to look at all groups just stay on All groups option.

*By pressing the button "Edit Graph Labels" you can write main and axis titles.

*Change the names of your Groups in the legend however you want by just clicks on them and write the names.

*In the next steps we need also the Luminescence data groups.

18 In "Group ration" select in the left the luminescence group and respectively in the right the OD group.

19 Press "Plot group ration over time vector" button to make the normalized luminescence group graph.

20 Repeat steps 18-19 for all respectively groups.

*If you make a mistake press the button "Clear Graph" and make the graph again.

*By pressing the button "Edit Graph Labels" you can write main and axis titles.

*Change the names of your Groups in the legend however you want by just clicks on them and write the names.

21 In "Group ration" select in the left the luminescence group and respectively in the right the OD group.

22 Press "2D Relation Plot (No Time Vector)" button to make the data of luminescence VS. OD group graphs.

*The next steps will perform actions in the "One-way ANOVA" in the top left tab in the software tab by click on him.

*In "One-way ANOVA" you have statistical analysis options.

23 In "Select groups (multi-select):" you can choose a groups to compare for making one way ANOVA statistical test.

24 After selecting the groups write your Factor name in the "Factor name (optional":(

25 Select time point that you want to do the compression between the groups .

26 Choose in "Post-hoc test:" from the 2 options, Tukey or Bonferroni.

27 Press "Run ANOVA" button to make the ANOVA groups graph.

28 Press "Show post-hoc plot" button to make the post-hoc plot.

29 Press "Clear ANOVA" button to delete the ANOVA groups graph.

*By pressing the button "Export results table" you can download the results.
