# Supplementary material for: Automated Platform for the Analysis of Multi-Plate Growth and Reporter Data
Source: Microorganisms. 2025 Aug 13;13(8):1889. doi: 10.3390/microorganisms13081889 (PMC12388276; doi:10.3390/microorganisms13081889)
Supplement: Supplementary file 1 [file microorganisms-13-01889-s001.zip › supporting 02.pdf]

Script download from github instructions:

1. Go to this URL: https://github.com/dorkain22/GROOT/blob/main/GROOT_ver14_1.m
2. Click the **“Download raw file”** button (next to or under the “Raw” button).
3. Your browser will automatically download the file named GROOT_ver14_1.m.
4. Move the downloaded file to your desired folder and open it with **MATLAB**.
